# Identification and molecular characterization of the nicotianamine synthase gene family in bread wheat

**DOI:** 10.1111/pbi.12577

**Published:** 2016-06-20

**Authors:** Julien Bonneau, Ute Baumann, Jesse Beasley, Yuan Li, Alexander A. T. Johnson

**Affiliations:** ^1^ School of BioSciences The University of Melbourne Melbourne Vic. Australia; ^2^ Australian Centre for Plant Functional Genomics The University of Adelaide Adelaide SA Australia

**Keywords:** phytosiderophore, iron, *Triticum aestivum*, gene expression, nutrient and metal transport, ion transport

## Abstract

Nicotianamine (NA) is a non‐protein amino acid involved in fundamental aspects of metal uptake, transport and homeostasis in all plants and constitutes the biosynthetic precursor of mugineic acid family phytosiderophores (MAs) in graminaceous plant species. Nicotianamine synthase (NAS) genes, which encode enzymes that synthesize NA from S‐adenosyl‐L‐methionine (SAM), are differentially regulated by iron (Fe) status in most plant species and plant genomes have been found to contain anywhere from 1 to 9 NAS genes. This study describes the identification of 21 NAS genes in the hexaploid bread wheat (*Triticum aestivum* L.) genome and their phylogenetic classification into two distinct clades. The *TaNAS* genes are highly expressed during germination, seedling growth and reproductive development. Fourteen of the clade I NAS genes were up‐regulated in root tissues under conditions of Fe deficiency. Protein sequence analyses revealed the presence of endocytosis motifs in all of the wheat NAS proteins as well as chloroplast, mitochondrial and secretory transit peptide signals in four proteins. These results greatly expand our knowledge of NAS gene families in graminaceous plant species as well as the genetics underlying Fe nutrition in bread wheat.

## Introduction

Nicotianamine (NA) is a non‐protein amino acid found in all higher plants (Noma and Noguchi, 1976; Noma *et al*., 1971). Nicotianamine was originally described as the ‘normalizing factor’ for tomato (*Lycopersicon esculentum* L) mutant *chloronerva* (Budesinsky *et al*., 1980). The *chloronerva* plants displayed retarded growth and signs of iron (Fe) deficiency despite containing high concentrations of Fe and other heavy metals in vegetative tissues, symptoms which were reversed upon exogenous application of NA. These findings, combined with three‐dimensional studies of NA structure, indicated that NA was crucial for *in planta* chelation, transport and homeostasis of Fe and other heavy metals (Budesinsky *et al*., 1980; Scholz *et al*., 1985). Later studies confirmed important roles for NA in short‐ and long‐distance transport of ferrous Fe (Fe^2+^) and other divalent metal cations such as zinc (Zn^2+^), manganese (Mn^2+^), copper (Cu^2+^) and nickel (Ni^2+^) as well as ferric iron (Fe^3+^) (Scholz *et al*., 1985; Stephan and Scholz, 1993; von Wiren *et al*., 1999).

Synthesis of NA occurs via trimerization of S‐adenosyl‐L‐methionine (SAM) in a process catalysed by nicotianamine synthase (NAS) enzymes (Higuchi *et al*., 1994). Nicotianamine is further converted to mugineic acid family phytosiderophores (MAs), such as 2′‐deoxymugineic acid (DMA), specifically in graminaceous plant species that utilize Strategy II Fe uptake (Marschner and Romheld, 1994; Marschner *et al*., 1986). Strategy II Fe uptake has been characterized in a number of graminaceous crops including rice (*Oryza sativa* L.), barley (*Hordeum vulgare* L.), maize (*Zea mays* L.) and wheat (*Triticum aestivum* L.) and involves the release of MAs into the rhizosphere, particularly under plant Fe deficiency, for chelation of Fe^3+^ and other essential transition metals (Marschner *et al*., 1986). Following chelation of Fe^3+^ with DMA or other MAs, the MAs‐Fe^3+^ complexes are reabsorbed through yellow stripe‐like (YSL) transporters at the root surface (Curie *et al*., 2001). The release of MAs facilitates the growth of Strategy II plant species on calcareous soils where high pH impedes the Fe reductive approach utilized by Strategy I plants (Guerinot and Yi, 1994; Romheld *et al*., 1982; Scholz *et al*., 1992).

Studies of the NAS genes and proteins of rice, barley and maize have revealed significant differences in NAS gene number and expression level as well as NAS protein structure, length and function across the three species. Three NAS genes are present in the rice genome (hereafter referred to as *OsNAS* genes), consisting of the highly homologous *OsNAS1* and *OsNAS2* genes on chromosome Os3 and the *OsNAS3* gene on chromosome Os7 (Inoue *et al*., 2003). The presence of 9‐10 NAS genes in the maize genome (hereafter referred to as *ZmNAS* genes) located on chromosomes 1, 7 and 9 represents a gene family three times larger than that of rice (Mizuno *et al*., 2003; Zhou *et al*., 2013a,b). The ZmNAS proteins vary between 327 and 601 amino acid (aa) in length and phylogenetic comparison to the NAS proteins of rice (three proteins), barley (nine proteins), *Arabidopsis thaliana* (three proteins) and *Solanum lycopersicum* (one protein) identified two clades or classes of NAS proteins within the Gramineae; these are referred to as clade I and II NAS proteins in this paper (Inoue *et al*., 2003; Mizuno *et al*., 2003; Perovic *et al*., 2007; Zhou *et al*., 2013a,b). The pairs of clade I NAS genes in maize are believed to result from chromosomal block duplication (Wei *et al*., 2007). Such duplication of NAS genes in rice and barley has also been described (Higuchi *et al*., 2001; Perovic *et al*., 2007). Expression of the clade I NAS genes in rice and maize is root specific, induced ubiquitously in root and shoot tissues under Fe deficiency and decreased under conditions of Fe excess; these findings suggest that clade I NAS genes function primarily in the regulation of NA and MAs biosynthesis, Fe uptake and long‐distance Fe translocation. Uniquely to maize, a pair of expressed clade I NAS genes (*ZmNAS2;1*/*2;2*) encodes NAS proteins approximately twice as long (601 aa) as the clade I NAS proteins found in rice and barley; however, enzymatic activity of these maize NAS proteins has not been detected (Mizuno *et al*., 2003; Zhou *et al*., 2013b). Expression of the clade II NAS genes in rice and maize is shoot specific, decreased and/or relocalized to root tissues under Fe deficiency and induced ubiquitously in root and shoot under conditions of Fe excess. These expression patterns suggest that clade II NAS proteins do not contribute to MAs biosynthesis under Fe deficiency and are instead primarily involved in NA biosynthesis for Fe loading of vascular tissues and maintenance of cellular Fe homeostasis.

Evidence suggests that clade I and II NAS proteins are targeted to various compartments within the plant cell; a recent bioinformatics study of the ZmNAS protein sequences revealed signal peptides localizing ZmNAS5 (clade II) and ZmNAS6;1 (clade I) to the mitochondria and chloroplasts, respectively (Zhou *et al*., 2013a). In rice, the OsNAS2 (clade I) protein contains two motifs (YXXФ and LL) involved in vesicular transport and OsNAS2 enzyme activity was found to be localized to rice root vesicles under Fe deficiency (Nozoye *et al*., 2014).

The presence of 9‐10 NAS genes in the barley genome (hereafter referred to as *HvNAS* genes) represents a gene family similar in size to that of maize (Herbik *et al*., 1999; Higuchi *et al*., 1999a,b, 2001; Mizuno *et al*., 2003; Perovic *et al*., 2007). The *HvNAS* genes have been classified into three synteny‐based groups through comparison with the rice genome (Perovic *et al*., 2007). Group 1 consists of the *HvNAS2*,* HvNAS3* and *NASHOR1a* genes and is located on barley chromosome 4H. The *NASHOR1a* gene is orthologous and syntenic to *OsNAS1* and *OsNAS2* on rice chromosome Os3. Group 2 contains the *NASHOR2* gene which is orthologous and syntenic to *OsNAS3* on rice chromosome Os7. Group 3 consists of five *HvNAS* genes that are nonsyntenic with rice. Four of the group 3 genes are located on chromosome 6H (*HvNAS1*,* HvNAS4*,* HvNAS7*,* NASHOR1b*) while *HvNAS5* is located on chromosome 2HS; these *HvNAS* genes are considered unique to the barley genome and are thought to have arisen from ectopic *HvNAS* gene duplication events (Perovic *et al*., 2007). In contrast to rice and maize, all *HvNAS* genes investigated to date show Fe deficiency inducible expression specifically in root tissues with no expression under Fe sufficiency nor in shoot tissues under any Fe status (Higuchi *et al*., 2001). The HvNAS proteins vary between 267 and 340 aa in length and little is known about their cellular localization.

While the NAS gene families of rice, maize and barley have been described in publications for over 10 years, there is only one publication to date (Pearce *et al*., 2014) identifying two genes belonging to the NAS gene family of bread wheat (*Triticum aestivum*), hereafter referred to as the *TaNAS* gene family. The paucity of information about *TaNAS* genes is surprising in the light of recent publications identifying NA and/or DMA as the predominant chelators of Fe in wheat white flour (Eagling *et al*., 2014a,b). In this paper, we describe the identification and characterization of a large *TaNAS* gene family in bread wheat using cv. Chinese Spring—the reference genome of the International Wheat Genome Sequence Consortium—and high‐yielding Australian cv. Gladius as our plant materials.

## Results

### Identification of 21 *TaNAS* genes located on multiple bread wheat chromosomes and subgenomes

**Figure 1:**
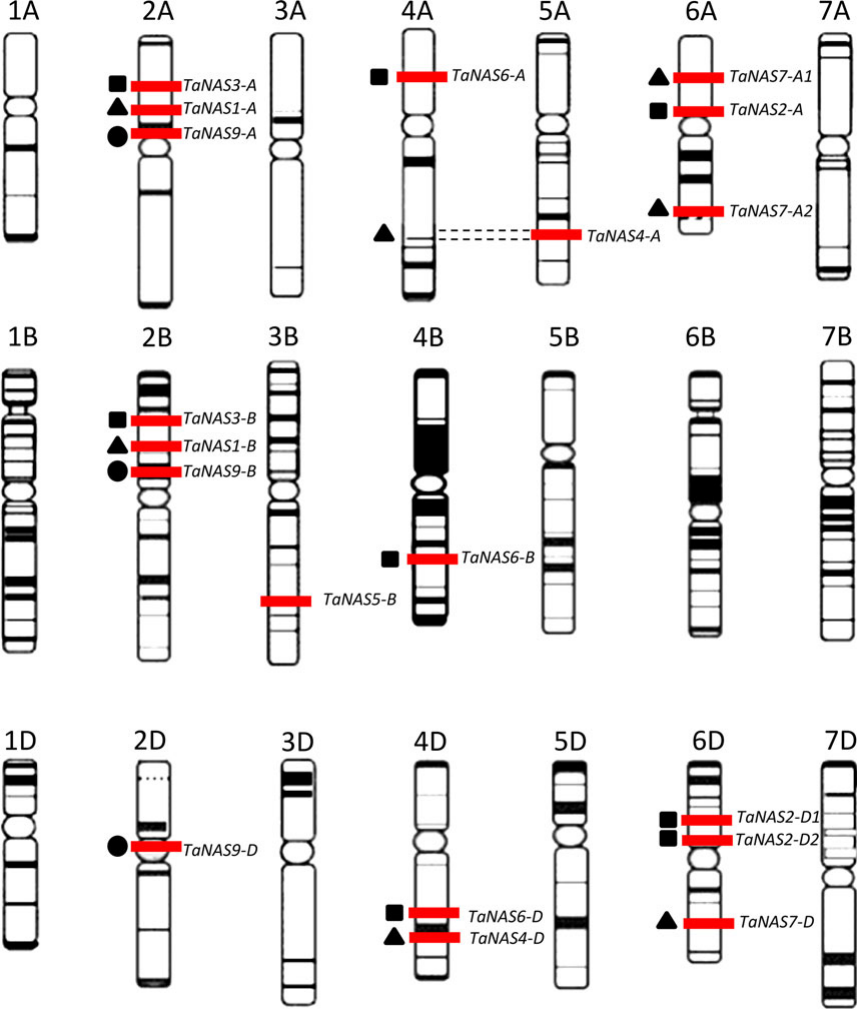
Distribution of 19 *TaNAS* genes across the seven chromosome groups and A, B and D subgenomes of bread wheat. Genes are represented by red bars and homeologous genes within chromosome groups, when present, are indicated by common symbol (■▲●). Dotted lines represent the 4AL‐5AL chromosomal translocation. Figure adapted from the IWGSC website (http://www.wheatgenome.org/).

We identified a total of 21 unique *TaNAS* genes and determined the genomic location of 19 of these genes on chromosome groups 2, 3, 4, 5 and 6 (Figure 1, Table 1). Several sets of homeologous *TaNAS* genes were identified on chromosome groups 2, 4 and 6 (Figure 1, Table S5). Chromosome group 2 contains three homeologous *TaNAS* genes located on the A, B and D subgenomes (*TaNAS9‐A*/*TaNAS9‐B*/*TaNAS9‐D*) and two pairs of homeologous *TaNAS* genes located only on the A and B subgenomes (*TaNAS3‐A*/*TaNAS3‐B* and *TaNAS1‐A*/*TaNAS1‐B*). Chromosome group 4 contains three homeologous *TaNAS* genes located on the A, B and D subgenomes (*TaNAS6‐A*/*TaNAS6‐B*/*TaNAS6‐D*). The homeologous *TaNAS4‐D* and *TaNAS4‐A* genes on chromosome groups 4 and 5 were likely separated by the known 4AL‐5AL wheat chromosome translocation (Hernandez *et al*., 2012). Chromosome group 6 contains three homeologous *TaNAS* genes located on the A and D subgenomes (*TaNAS2‐A*/*TaNAS2‐D1*/*TaNAS2‐D2*) and the two 6DS genes share 98.5% genomic sequence identity. Chromosome group 6 also contains an additional three homeologous *TaNAS* genes located on the A and D subgenomes (*TaNAS7‐A1*/*TaNAS7‐A2*/*TaNAS7‐D*) and the 6AS and 6DL genes share 96.2% genomic sequence identity. We identified one *TaNAS* gene on chromosome group 3 without any homeologues (*TaNAS5‐B*).

**Table 1:** Identification of 21 *TaNAS* genes in bread wheat. Each *TaNAS* gene was assigned a unique name (first column) based on homeologous grouping and subgenome A, B or D (U indicates unknown). An additional number was added to the gene name when more than one NAS gene belonging to the same homeologous group was located on the same chromosome. The third and fourth columns provide the IWGSC contig (https://urgi.versailles.inra.fr/blast/blast.php) on which the gene was identified and the MIPS database gene annotation (http://pgsb.helmholtz-muenchen.de/plant/index.jsp) for each of the *TaNAS* genes. The sequences of PCR primers used to amplify each gene from genomic DNA, as well as the expected PCR product sizes (bp), are provided in columns 5–7

Gene name	Chromosome location	IWGSC contig	MIPS database gene annotation	Forward primer sequence 5′ to 3′	Reverse primer sequence 5′ to 3′	PCR product length
*TaNAS1‐A*	2AS	ctg5260464	Traes_2AS_1FB8F6760.1	TCAAGCATCAGCTCCATCAT	AGGCTCCCCCTTTTCTTCA	973
		ctg5186953	Traes_2AS_D50EEDA84.1	–	–	–
*TaNAS1‐B*	2BS	ctg5223571	Traes_2BS_A984C71A6.1	TCAAGCATCAGCTCCATCAA	ATGTGCGGGGAATCATCTAC	1026
*TaNAS2‐A*	6AS	ctg4374229	Traes_6AS_15ABF3B3E1.1	ATCAGCACGCACATTTTCAA	GGTCACCAAGAAGCAACGAT	1025
		ctg4418337	Traes_6AS_89FA598FB.1	–	–	–
*TaNAS2‐D1*	6DS	ctg2123013	Traes_6DS_1965FC73E.1	CACATTTTCTCCTGCTTCCT	ATGATCGGCACGCACATTTCG	1067
*TaNAS2‐D2*	6DS	ctg2092960	Traes_6DS_92693EC0A.1	GAGATGATCAGCACGCACAT	CGGTCACCAAGAAGCAATTA	1041
*TaNAS3‐A*	2AS	ctg5220125	Traes_2AS_F55243E0C.1	AGCTCGATCAACCACTCTCC	GCACATGCACAACCACTACC	1089
*TaNAS3‐B*	2BS	ctg5244458	Traes_2BS_98815CE06.1	AGCTCCATCAACCACTCTCT	GCACATGCACAACCACTACT	1091
*TaNAS4‐A*	5AL	ctg624885	Traes_5AL_8715BE19D.1	GCCTGCACTGAGGTACCAAC	GATCAAGCAGCGAGGTAGGA	1099
*TaNAS4‐U*	U	–	–	CATGGATGCACACCTTTTGT	CTTGGCAACGATCGATCAGAAGA	1056
*TaNAS4‐D*	4DL	ctg14334745	Traes_4DL_4E10A6DFB.1	CATGGATGCACACCTTTTGT	CAATTACCACGTGTGGTTGC	1220
*TaNAS5‐B*	3BU	ctg10414150	Traes_3B_3AC6B469E.1	CGAGCCTTATTGGGAGTGTC	GACCACACATGCACACGTTC	1205
		ctg10751094	Traes_3B_A52E3C298.1	–	–	–
*TaNAS6‐A*	4AS	ctg6009291	Traes_4AS_34072BFC9.1	GGCATGAAACCAGCCATATT	AGCAGCCACGTATGGATGAG	2392
*TaNAS6‐B*	4BL	ctg6980195	Traes_4BL_BB0FC3BD3.1	TGGTGCACGCTCTTTATTCG	ACGTACGGACGATGACCAC	1489
*TaNAS6‐D*	4DL	ctg14432849	Traes_4DL_AF0869DDB.1	TGGTGCAGGCTCTTTATTCA	ACGTACTGATGATGACCGC	1361
*TaNAS7‐A1*	6AS	ctg4338157	Traes_6AS_5D0B8CF891.1	GGTACCAAGGCAAGAACACAC	GATGATGACCTCGCAGTCG	1043
*TaNAS7‐A2*	6AL	ctg5810064	Traes_6AL_F4AEC0314.1	TTCCATAGCTCATCAAGCAA	AACTCCTCTCTCTTCTGGGTCA	1070
*TaNAS7‐D*	6DL	ctg3268810	Traes_6DL_1470C4162.1	CATTAAAATGGACGCCCAGA	GCACGGATGATGACCTCAC	1024
*TaNAS8‐U*	U	–	Traes_XX_432D1C804.1	–	–	–
*TaNAS9‐A*	2AS	ctg5303755	Traes_2AS_DEDC612AE.1	AGCTAGCTAGTGCCCTCTGC	CATGTATGTATCGGTCGGTGA	1221
		ctg5195617	Traes_2AS_452FED53F.1	–	–	–
*TaNAS9‐B*	2BS	ctg5246738	Traes_2BS_CB79BAFB1.1	CTCACTCTCAGAGCCCCTCA	TCCATGCATGAGGACAAACG	1263
*TaNAS9‐D*	2DS	ctg5343018	Traes_2DS_FE40FC64C.1	CAATTAGCAGCTGATCTGTCG	TCCATGCATGAGGACAAGC	1182
		ctg2341167	–	–	–	–

**Figure 2:**
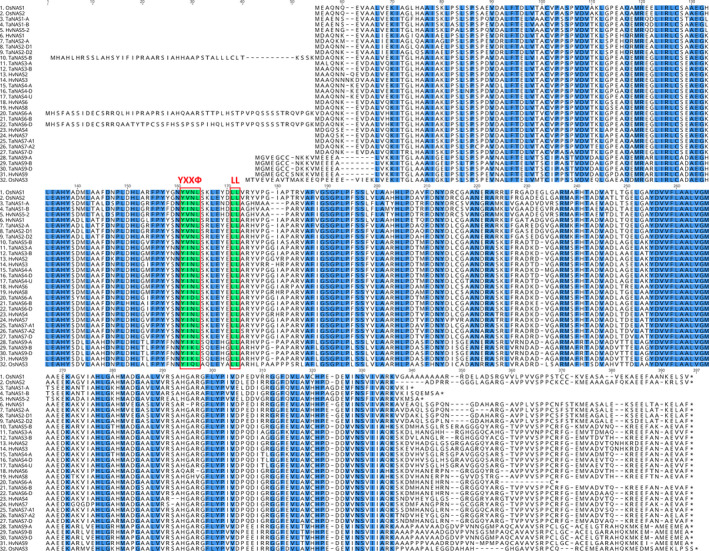
Amino acid sequence alignment of the NAS proteins from wheat (TaNAS), rice (OsNAS) and barley (HvNAS) identifies conserved regions and motifs. Blue shading corresponds to 100% conservation of amino acids between species. The YXXФ and LL motifs of the NAS superfamily protein are shaded in green and outlined in red (Y = tyrosine; X = any amino acid residue; Ф = amino acids with bulky hydrophobic residues; LL = di‐leucine).

The genomic location of two *TaNAS* genes (*TaNAS4‐U* and *TaNAS8‐U*) could not be determined based on currently available wheat genome sequence. The genomic sequence of *TaNAS4‐U* shares over 95% nucleic acid identity with the *TaNAS4‐A* and *TaNAS4‐D* genes and most likely represents the homeologous gene located on subgenome B. The *TaNAS4‐U* gene could only be amplified from cv. Gladius DNA, suggesting that the gene is present in only one of the investigated wheat cultivars or has significant polymorphisms between the two wheat cultivars. The partial (168 bp) sequence obtained for the *TaNAS8‐U* gene shares 94.6% nucleic acid identity with the two homeologous *TaNAS3‐A* and *TaNAS3‐B* genes located on wheat chromosome group 2 and likely represents the homeologous gene *TaNAS3‐D* located on subgenome D. Overall, the 20 full‐length *TaNAS* genes identified in this study share medium to high identity in terms of nucleic acid sequence (67.8%–98.5%) as well as medium to high similarity in protein sequence (57.8%–99.4%) based on chemical reactivity of amino acid side chains (Figure 2).

### The *TaNAS* genes separate into two distinct clades and show high similarity to *HvNAS* genes

**Figure 3:**
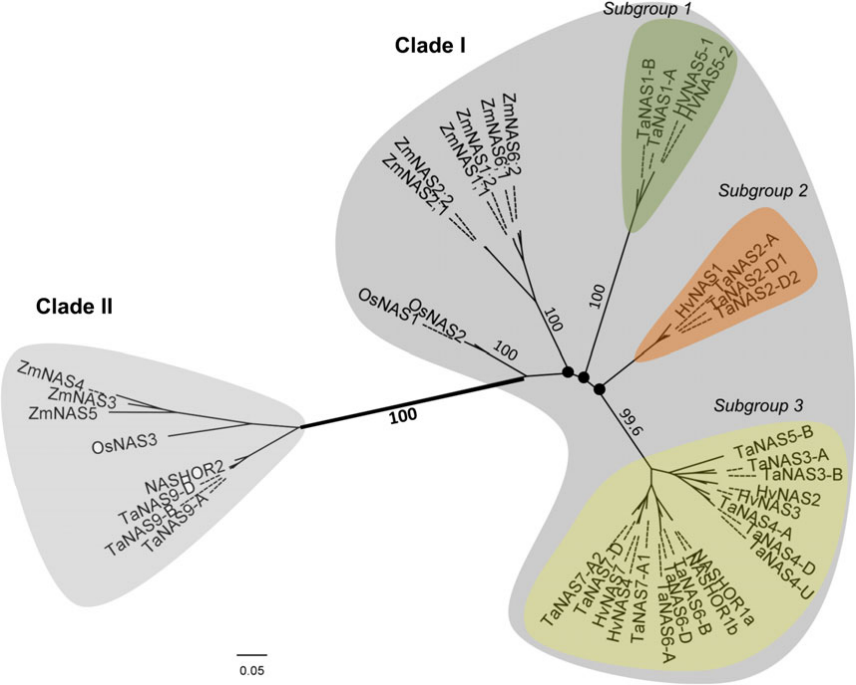
Phylogenetic relationship of the wheat, rice, barley and maize NAS genes. An unrooted tree of NAS gene coding sequences from wheat (*TaNAS*), rice (*OsNAS*), barley (*HvNAS*) and maize (*ZmNAS*) identifies the clade I and II NAS genes of these species. Clade I separates into three subgroups comprising only wheat and barley genes. Black nodes (●) represent weak bootstrap values (<75%). The scale bar corresponds to branch length and longer branches correspond to greater numbers of nucleic acid polymorphisms along the sequence.

Our phylogenetic analyses identified two distinct clades of *TaNAS* genes that share most similarity with barley *HvNAS* genes (Figure 3). Clade I contains 17 of the *TaNAS* genes as well as the previously described clade I NAS genes of rice, maize and barley; clade I further separates into three subgroups with bootstrap proportions of <75%. Phylogenetic analyses revealed that the *TaNAS* and *HvNAS* genes within each clade I subgroup are most closely related with bootstrap proportions of 99.6%–100%. Clade I subgroup 1 contains two *TaNAS* genes from wheat chromosome groups 2A and 2B (*TaNAS1‐A* and *TaNAS1‐B*) and the orthologous barley gene *HvNAS5‐1/2* located on chromosome 2HS. Clade I subgroup 2 contains three *TaNAS* genes from wheat chromosome groups 6A and 6D (*TaNAS2‐A*,* TaNAS2‐D1* and *TaNAS2‐D2*) and the orthologous barley gene *HvNAS1* located on chromosome 6HS. Clade I subgroup 3 is the largest of the subgroups and comprises 12 *TaNAS* genes located on various wheat chromosome groups and six orthologous *HvNAS* genes. Clade II is a much smaller NAS gene clade with no subgroups and contains three homeologous *TaNAS* genes located on wheat chromosome group 2 (*TaNAS9‐A*/*TaNAS9‐B*/*TaNAS9‐D*) as well as the previously described clade II NAS genes of rice, maize and barley.

### The TaNAS proteins show variation in the length of N‐ and C‐terminal regions and are targeted to several subcellular locations

Alignment of the protein sequences encoded by the 20 full‐length *TaNAS* genes showed that TaNAS proteins vary in length from 287 to 385 amino acids and that several regions of the proteins are highly conserved (Figure 2). Two of the proteins have uncharacteristically long N‐terminal regions that contain either mitochondrial targeting or chloroplast transit peptides (TaNAS5‐B and TaNAS6‐D), two others have uncharacteristically short C‐terminal regions (TaNAS1‐A and TaNAS1‐B), while one protein has both an uncharacteristically long N‐terminal containing a chloroplast transit peptide and a short C‐terminal region (TaNAS6‐A). The TaNAS2‐A protein has an N‐terminal secretory pathway signal peptide (Table S4). All of the TaNAS proteins contain YXXФ (Ф = amino acids with bulky hydrophobic residues) and di‐leucine (LL) motifs in the N‐terminal region that have previously been described as characteristic features of NAS superfamily proteins in plants and indicate that they are localized to cytoplasmic vesicles (Figure 2).

### Clade I and II *TaNAS* genes are expressed during germination, seeding growth and reproductive development

**Figure 4:**
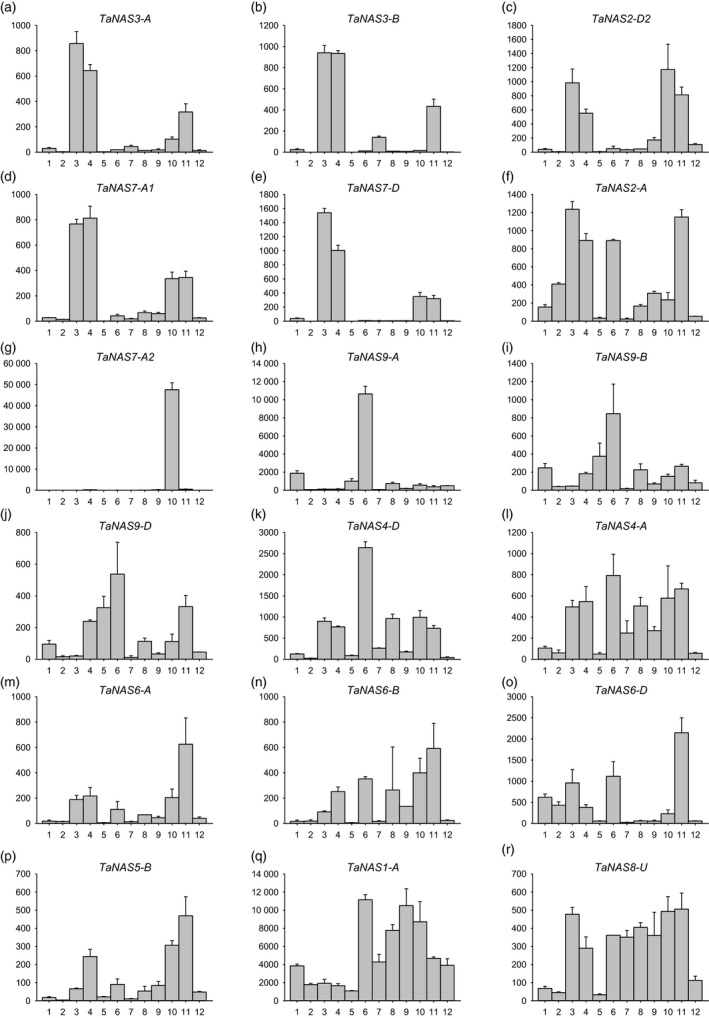
Relative expression of 18 *TaNAS* genes in 12 different tissues and developmental stages of bread wheat cv. Chinese Spring. Relative expression of *TaNAS* genes is provided for the following: (1) embryo 1 DAS; (2) coleoptile 2 DAS; (3) radicle 2 DAS; (4) seedling root 10‐12 DAS; (5) seedling crown 10–12 DAS; (6) seedling leaf 10–12 DAS; (7) immature inflorescence; (8) bracts; (9) pistil; (10) anthers; (11) caryopsis 3‐5 DAP; (12) embryo 22 DAP. Units on the *y*‐axis indicate normalized mRNA copies per μg of total RNA. Error bars indicate SEM of three technical replicates derived from one bulked biological replicate.

Eighteen of the 21 *TaNAS* genes were expressed in young root tissues, seedling leaf and/or developing reproductive organs of cv. Chinese Spring (Figure 4). In most cases, homeologous *TaNAS* genes displayed similar expression profiles; however, in some instances specific homeologous genes were either more tissue specific or higher in expression relative to the corresponding homeologues. The homeologous *TaNAS3‐A*/*TaNAS3‐B* genes as well as the *TaNAS7‐A1, TaNAS7‐D* and *TaNAS2‐D2* genes were most highly expressed in the embryonic radicle 2 days after sowing (DAS) and seedling root 10‐12 DAS (Figures 4a‐e). The *TaNAS2‐A* gene (Figure 4f), a homeologous gene to *TaNAS2‐D2*, was not only highly expressed in young root tissues but also in seedling leaf and reproductive organs with highest expression in the caryopsis tissue 3–5 days after pollination (DAP). The *TaNAS7‐A2* gene, a homeologous gene to *TaNAS7‐D*, displayed highly anther‐specific expression with approximately 100‐fold higher expression in anther tissue relative to all other organs (Figure 4g). The homeologous *TaNAS4‐D*/*TaNAS4‐A* genes and the homeologous clade II *TaNAS9‐A*/*TaNAS9‐B*/*TaNAS9‐D* genes were all most highly expressed in leaf tissues (Figure 4h‐l). Expression of the subgenome A *TaNAS9‐A* gene was 10‐fold higher than the corresponding subgenome B and D homeologues (*TaNAS9‐B* and *TaNAS9‐D*). The *TaNAS5‐B* gene as well as the homeologous *TaNAS6‐A*/*TaNAS6‐B*/*TaNAS6‐D* genes had generally low expression levels with most expression detected in the caryopsis tissue 3–5 DAP (Figure 4l‐p). Expression of the subgenome D *TaNAS6‐D* gene was threefold higher than the corresponding subgenome A and B homeologues (*TaNAS6‐A* and *TaNAS6‐B*). The *TaNAS1‐A* and *TaNAS8‐U* genes had the most ubiquitous expression profiles of the *TaNAS* genes with expression detected in most tissue types and developmental stages (Figure 4q‐r). No expression of the *TaNAS1‐B*,* TaNAS2‐D1* and *TaNAS4‐U* genes was detected in cv. Chinese Spring.

### Clade I *TaNAS* genes are expressed in root tissues and up‐regulated under iron deficiency during vegetative growth

**Figure 5:**
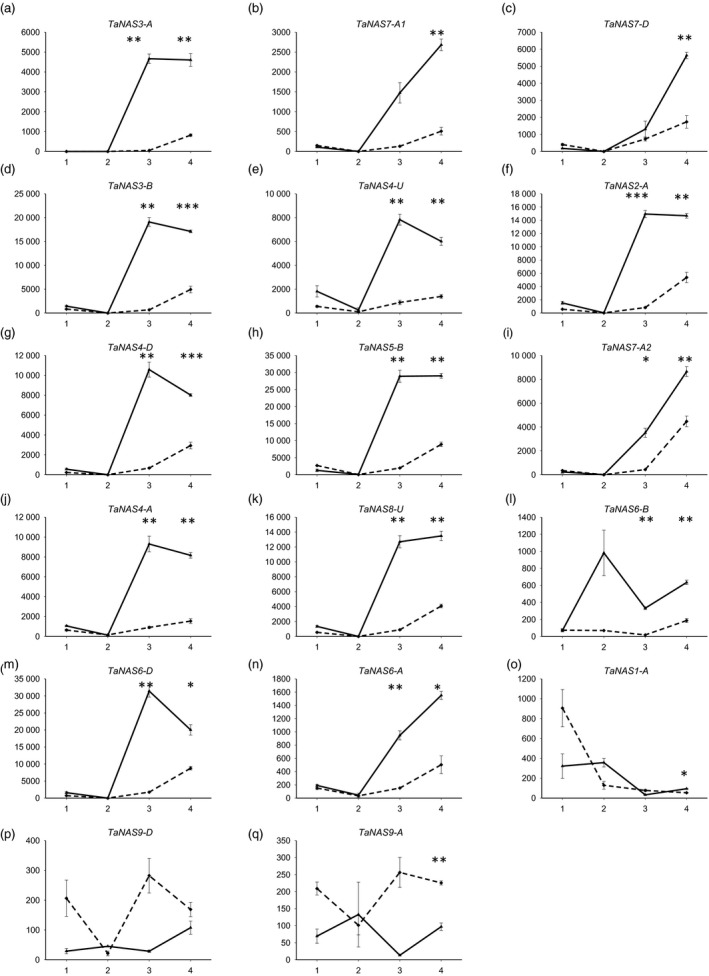
Relative expression of 17 *TaNAS* genes in root tissues of bread wheat cv. Gladius under iron‐sufficient/deficient conditions. Gene expression in plants grown under Fe‐sufficient (dashed line) or Fe‐deficient (solid line) conditions is presented at four time points of the 7 day treatment: day 0 (1), day 1 (2), day 5 (3) and day 7 (4). Units on the *y*‐axis indicate normalized mRNA copies per μg of total RNA. Error bars indicate SEM of three biological replicates for time points 1, 3 and 4 and two biological replicates for time point 2. Asterisks indicate statistically significant differences for the effect of condition (+Fe and –Fe) at each time point (one‐way ANOVA, Tukey's test,*P value ≤0.05; **P value ≤0.01; ***P value ≤0.001).

The analysis of *TaNAS* gene expression in the root and shoot of 4‐week‐old cv. Gladius plants found that nearly all of the clade I *TaNAS* genes were expressed in root tissues of the vegetatively growing bread wheat plants under conditions of Fe sufficiency and deficiency (Figure 5). Under Fe‐sufficient growth conditions (control treatment), 14 of the clade I *TaNAS* genes were expressed at low levels in root tissues until day 5 of the treatment period and then moderately expressed from days 5–7 (dashed lines in Figure 5a‐n). Increased levels of *TaNAS* gene expression towards the end of the control treatment period may reflect increased demands for NA‐ and/or DMA‐mediated Fe transport as wheat plants begin to rapidly grow and tiller at the 4‐week growth stage. Under Fe‐deficient growth conditions, expression of the 14 clade I *TaNAS* genes was significantly up‐regulated in root tissues with approximately 15‐fold higher expression at day 5 and threefold higher expression at day 7 of the treatment relative to controls (solid lines in Figure 5a‐n). Three additional *TaNAS* genes, including the clade II *TaNAS9‐A* and *TaNAS9‐D* genes, were differentially expressed under Fe deficiency in the root; however, their expression levels were significantly lower than that of the previously mentioned 14 genes and no clear expression patterns were identified (Figure 5o‐q).

Only seven *TaNAS* genes, including the three homeologous clade II *TaNAS* genes located on wheat chromosome group 2 (*TaNAS9‐A*/*TaNAS9‐B*/*TaNAS9‐D*), were expressed at low levels in cv. Gladius shoot tissues (Figure S3). The average level of *TaNAS* gene expression under both Fe‐sufficient and Fe‐deficient growth conditions in shoots was 30‐fold lower than that of roots and bordered on the detection limit for qRT‐PCR. Unlike the expression patterns observed in roots, Fe‐deficient growth conditions tended to decrease the already low *TaNAS* gene shoot expression levels. No expression of the *TaNAS1‐B*,* TaNAS2‐D1* and *TaNAS2‐D2* genes was detected in cv. Gladius.

## Discussion

The 21 *TaNAS* genes located on the A, B and D subgenomes of bread wheat represent the largest plant NAS gene family identified to date. Most members of this large gene family are transcriptionally active at multiple stages of bread wheat growth and development as well as under conditions of Fe deficiency. The results obtained from our molecular characterization of the *TaNAS* gene family allow several conclusions to be made about the origin of *TaNAS* genes as well as their function within the wheat plant.

### Many clade I and II *TaNAS* genes share a common origin with *HvNAS* genes

Our study identified two distinct clades of *TaNAS* genes, a result that is consistent with previous studies of maize, barley and rice NAS genes (Perovic *et al*., 2007; Zhou *et al*., 2013b). The separation into the two clades is supported by high bootstrap values and branch lengths. Seventeen of the 20 full‐length *TaNAS* genes belong to clade I, and in our phylogenetic analyses, these genes clustered as three subgroups comprising only wheat and barley NAS genes. This suggests that each *Triticeae* subgroup is derived from distinct ancestors.

Clade I subgroups 1 and 2 contain two groups of homeologous *TaNAS* genes—the *TaNAS1‐A*/*TaNAS1‐B* genes located on chromosome group 2 and the *TaNAS2‐A*/*TaNAS2‐D1*/*TaNAS2‐D2* genes located on chromosome group 6—which are syntenic with the duplicated *HvNAS5‐1/2* and *HvNAS1* genes located on barley chromosomes 2 and 6, respectively (Perovic *et al*., 2007). It was originally hypothesized that a barley‐specific NAS gene duplication event gave rise to the *HvNAS5‐1* and *HvNAS1* genes; however, our results indicate that this gene duplication event likely occurred in a diploid wheat–barley ancestor.

Clade I subgroup 3 contains 12 *TaNAS* and 6 *HvNAS* genes and our results suggest that there is macro‐synteny of NAS genes between wheat and barley in this subgroup. In addition, our results indicate that intra‐ and interchromosomal NAS gene duplication events gave rise to this large subgroup of NAS genes in both wheat and barley. The homeologous *TaNAS4‐D*/*TaNAS4‐A* genes located on wheat chromosome groups 4 and 5 and the homeologous *TaNAS7‐A2*/*TaNAS7‐D* genes located on wheat chromosome group 6 are orthologous and syntenic to the *HvNAS2/HvNAS3* and *HvNAS4/HvNAS7* intrachromosomal gene duplication pairs located on barley chromosomes 4HL and 6HL, respectively (Perovic *et al*., 2007). The homeologous *TaNAS6‐A*/*TaNAS6‐B*/*TaNAS6‐D* genes located on wheat chromosome group 4 are orthologous and syntenic to the *NASHOR1a* gene located on barley chromosome 4HL. The putatively homeologous *TaNAS3‐A*/*TaNAS3‐B*/*TaNAS8‐U* genes are not orthologous nor syntenic to any of the barley NAS genes, indicating that wheat‐specific NAS gene duplication events most likely occurred in a diploid wheat ancestor to give rise to these genes. Similar wheat‐specific gene duplication events and/or chromosomal translocations are also likely responsible for origin of the *TaNAS7‐A1*,* TaNAS2‐D1*,* TaNAS2‐D2*,* TaNAS4‐U* and *TaNAS5‐B* genes. Interestingly, the *TaNAS4‐U* and *TaNAS5‐B* genes of clade I subgroup 3 contain significant differences between cvs. Gladius and Chinese Spring, suggesting that some genes within this subgroup are evolving rapidly. The *TaNAS4‐U* gene may be specific to cv. Gladius or have significant polymorphisms in cv. Chinese Spring as it could not be amplified from genomic DNA nor cDNA of cv. Chinese Spring. The *TaNAS5‐B* gene, which has no homeologues on the A and D subgenomes, is functional in cv. Gladius yet contains a SNP (G→C) in the cv. Chinese Spring coding sequence that creates a premature stop codon (Figure S4).

Clade II contains the homeologous *TaNAS9‐A*/*TaNAS9‐B*/*TaNAS9‐D* genes that are orthologous and syntenic to the barley *NASHOR2* gene, orthologous to the rice *OsNAS3* gene and orthologous to the maize *ZmNAS3*,* ZmNAS4* and *ZmNAS5* genes (Figure 3). In contrast to the clade I *TaNAS* genes, there is no evidence for gene duplication, translocation or deletion in the origin of the clade II *TaNAS* genes.

### The *TaNAS* gene expression profiles indicate key roles for nicotianamine in bread wheat germination, seedling growth, reproductive development and iron deficiency response

The finding that several *TaNAS* genes are expressed during seed germination and seedling growth (namely in the embryonic radicle, seedling root and seedling shoot) indicates a requirement for NA biosynthesis at early stages of bread wheat growth and development. The rice *OsNAS* genes are also expressed at early stages of rice seed germination (Nozoye *et al*., 2007). We can hypothesize that NA functions as an important chelator of divalent metal cations such as Fe^2+^ and Zn^2+^ during wheat germination and seedling growth and helps to redistribute these metals at the tissue/cellular level and maintain metal homeostasis. Nicotianamine is also the biosynthetic precursor to mugineic acid family phytosiderophores such as DMA that wheat plants secrete from root tissues in order to absorb rhizospheric Fe^3+^ and it is likely that a significant proportion of wheat seedling root NA is diverted towards DMA biosynthesis. In general, the clade I *TaNAS* genes appear to play a major role in root‐specific NA production at the germination and seedling stages while the clade II *TaNAS* genes contribute more towards shoot‐specific NA production at these stages. Similar expression trends have been observed for the clade I and II NAS genes of rice and maize (Inoue *et al*., 2003; Zhou *et al*., 2013b).

Expression of several *TaNAS* genes in tissues of the bread wheat inflorescence, particularly the anther, and in developing grain indicates that NA is essential for bread wheat reproduction and complements the results of biochemical studies that have identified NA and/or DMA as predominant chelators of Fe in bread wheat white flour (Eagling *et al*., 2014a,b; Walker and Waters, 2011). Anthers are known to be major sink tissues for Fe in Arabidopsis and high expression of several *TaNAS* genes in the wheat anther, in particular the anther‐specific *TaNAS7‐A2* gene, suggests that wheat anthers are major Fe sinks and that NA is required to chelate Fe and protect against oxidative damage in this reproductive organ (Chu *et al*., 2010; Roschzttardtz *et al*., 2013; Takahashi *et al*., 2003). The finding that several *TaNAS* genes are expressed in the caryopsis 3‐5 DAP and the embryo 22 DAP likely reflects a requirement for NA to mobilize Fe and other divalent metals to the developing embryo. Expression of the rice *OsNAS1* and *OsNAS2* genes has also been detected in developing rice seed and the embryo (Dash *et al*., 2012; Fujita *et al*., 2010; Jain *et al*., 2007).

Nearly all of the clade I *TaNAS* genes were expressed at low to moderate levels in the roots of 4‐week‐old cv. Gladius plants grown under Fe sufficiency. These low levels of root‐specific *TaNAS* expression presumably provide sufficient NA to enable DMA biosynthesis as well as root to shoot translocation of Fe under sufficient conditions. Expression of the clade I *TaNAS* genes was significantly up‐regulated in root tissues under Fe deficiency and this result is consistent with the Fe deficiency response of barley where all of the *HvNAS* genes are significantly up‐regulated in root tissues in response to Fe deficiency (Higuchi *et al*., 2001). Increased expression of the clade I *TaNAS* genes in root tissues should result in greater quantities of NA (and DMA) to increase Fe uptake from the rhizosphere as well as Fe mobilization within the plant. The Fe deficiency response of rice differs somewhat from that of barley in that the rice clade I *OsNAS1* and *OsNAS2* genes are up‐regulated in both root and leaf tissues under Fe deficiency (Inoue *et al*., 2003). The lack of significant *TaNAS* gene expression in the shoot of vegetatively growing wheat plants under Fe deficiency indicates that the wheat Fe deficiency response is more similar to that of barley than rice and involves root‐specific induction of clade I *TaNAS* genes.

Our finding that, in some cases, *TaNAS* genes located on one subgenome were more highly expressed than corresponding homeologous genes on other subgenomes is a common feature of gene expression in hexaploid bread wheat. Genetic studies have found that approximately 55% of bread wheat genes in the three subgenomes display ‘unbalanced’ expression where at least one homeologous gene dominates. Silencing or loss of homeologous genes also frequently occurs in the bread wheat genome (Borg *et al*., 2012; Leach *et al*., 2014; Nussbaumer *et al*., 2015).

### The TaNAS proteins localize to cytoplasmic vesicles, chloroplasts and mitochondria within bread wheat cells and show variation in the length of N‐ and C‐terminal regions

The 20 full‐length TaNAS proteins identified in this study contain di‐leucine (LL) and YXXФ endocytosis motifs in the N‐terminal region as previously found in rice and barley proteins which suggests that the TaNAS proteins are enzymatically active and localized to cytoplasmic vesicles where NA biosynthesis is likely to take place (Figure 2, Geldner and Robatzek, 2008; Robert *et al*., 2005; Zuo *et al*., 2000). Rice OsNAS proteins also contain these motifs and substitution mutations in either motif of the OsNAS2 protein disrupt MAs vesicle formation and movement in rice root cells (Nozoye *et al*., 2014).

Three of the clade I TaNAS proteins have uncharacteristically long N‐terminal regions that were determined by *in silico* analyses to contain peptides for mitochondrial targeting (TaNAS5‐B) or chloroplast transit (TaNAS6‐A and TaNAS6‐D). We identified an EST corresponding to the long N‐terminal region of the *TaNAS6‐D* gene (GenBank identifier CA637385), indicating that these long N‐terminal regions are transcribed and that specific clade I TaNAS proteins are targeted to mitochondria and chloroplasts to facilitate NA biosynthesis and maintenance of Fe homeostasis within these organelles. Furthermore, the presence of a secretory signal peptide in the N‐terminal region of the TaNAS2‐A protein suggests that certain TaNAS proteins may be secreted from the cell to enable NA biosynthesis in other parts of the plant. Similar subcellular targeting of NAS proteins has been suggested in maize, where four of the nine ZmNAS proteins contain peptides for secretion or targeting to chloroplasts and mitochondria (Zhou *et al*., 2013a). The function of the short C‐terminal regions of the TaNAS1‐A and TaNAS1‐B proteins is currently unknown; however, a similar short C‐terminal region is present in the orthologous HvNAS5‐1 protein of barley (Figure 2).

### Applications towards plant breeding

The 21 *TaNAS* genes described in this study represent a valuable resource for plant breeders working to improve the growth, yield and nutritional value of bread wheat, a major food staple that provides 20% of the calories and protein consumed by humans worldwide. Iron toxicity often occurs when wheat plants are cultivated under waterlogged conditions, while Fe deficiency can occur in plants grown on calcareous soil. These abiotic stresses frequently lead to early leaf senescence and negatively impact on grain yield and quality. Altered expression of specific *TaNAS* genes could either increase or suppress the Fe deficiency response and thereby facilitate the development of bread wheat cultivars adapted to Fe‐rich or Fe‐poor growth conditions. Furthermore, increased activity of *TaNAS* genes with expression within the developing grain could lead to the production of more nutritious wheat varieties containing enhanced concentrations of bioavailable grain Fe.

## Experimental procedures

### Bread wheat cv. Gladius germination and hydroponic growth conditions

Seeds of cv. Gladius were germinated on moist filter paper for 5 days. Seedlings were transferred to 20‐L tubs (30 seedlings per tub) containing hydroponic growth solution as described in Genc *et al*. (2007) and grown in a University of Melbourne glasshouse (Parkville, Vic., Australia) for 4 weeks over January 2014 with average daily temperatures of 24.6 °C and 58.8% humidity. Hydroponic solutions were replaced every 2 days and pH was maintained between 6.5 and 7. Iron deficiency treatments commenced when plants were 3 weeks old; one tub of seedlings was grown for seven additional days in hydroponic solution lacking Fe, whereas a control tub of seedlings was grown for seven additional days in hydroponic solution with Fe. Three plants were individually harvested from the Fe‐deficient and control tubs at four time points—days 0 (experiment start), 1, 5 and 7—and the root and shoot tissues were snap‐frozen in liquid nitrogen. Prior to harvest, shoot weight and length and leaf SPAD value (Chlorophyll Meter SPAD‐502Plus, Konica Minolta, Europe) were recorded for each plant. Plants grown under Fe deficiency developed leaf Fe deficiency symptoms as indicated by significantly reduced chlorophyll content of the fourth fully expanded leaf on day 5 (*P* < 0.05) and the fifth fully expanded leaf on day 7 (*P* < 0.001) of the treatment (Figure S1).

### Identification and naming of the *TaNAS* genes

The *HvNAS* coding sequences (Table S1) were used as queries in BLAST searches against the 41 genomic databases of the International Wheat Genome Sequencing Consortium (IWGSC, 2014—http://www.wheatgenome.org) to identify sequences encoding putative *TaNAS* genes. Matching sequences from the IWGSC portal were annotated using the TriAnnot v1.4 (http://wheat-urgi.versailles.inra.fr) and FGENESH (http://www.softberry.com/) pipelines (Solovyev *et al*., 2006). In cases where a full gene sequence was not available from IWGSC, we further searched the *Triticum aestivum* cv. Chinese Spring 5× genome database (http://www.cerealsdb.uk.net). Gene sequences were merged and assembled using Geneious Pro 5.6.6 (Biomatters, http://www.geneious.com/). Each *TaNAS* gene was assigned a unique name based on homeologous grouping and subgenome and followed the recommended rules for gene symbolization in wheat (http://wheat.pw.usda.gov/ggpages/wgc/98/Intro.htm, Table 1). Gene names *TaNAS1‐A* and *TaNAS9‐A/TaNAS9‐B* correspond to the *TaNAS1* and *TaNAS3* genes, respectively, described in Pearce *et al*. (2014). Homeologous *TaNAS* genes were identified on the basis of their chromosome group, arm location and percentage of nucleic acid identity with a 94% identity threshold level for homeologous genes. The classification of homeologous genes also took into account the known 4AL‐5AL wheat chromosome translocation (Hernandez *et al*., 2012) and the pericentromeric inversion of wheat chromosome 4AS‐4AL (Liu *et al*., 1992).

Gene prediction models including predicted CDS and protein sequence were obtained for each of the identified *TaNAS* genes using FGENESH software (http://www.softberry.com). CDS alignments (CLUSTALW) and protein alignments based on the degree of amino acid conservation (CLUSTALW—cost matrix BLOSUM62, threshold 1) were performed in Geneious Pro 5.6.6. The TaNAS protein sequences were analysed for the presence of target peptides using the recommended protocol for TargetP 1.1 as described by Emanuelsson *et al*. (2007). Validation of the *TaNAS* genes was obtained by BLASTN searches of the 19 *TaNAS* coding sequences to the recently released TGACv1 genome assembly (http://pre.plants.ensembl.org/Triticum_aestivum/Info/Index), all of which returned a single TGAC scaffold with identity equal to or higher than 99%.

### Gene sequencing

Subgenome‐specific PCR primers for each *TaNAS* gene were designed using the respective IWGSC contig sequences and Primer3 software (Table 1; Untergasser *et al*., 2012); the primer pairs were used to amplify fragments from cvs. Gladius and Chinese Spring genomic DNA. The subgenome specificity of each primer pair was verified using cv. Chinese Spring nulli‐tetrasomic DNA (Sears, 1954). PCR amplification cycles consisted of 1 cycle = 1 min 95 °C; 35 cycles = 20 s 95 °C, 30 s 61–62 °C, 1 min 72 °C; 1 cycle = 5 min 72 °C. PCRs were performed in a final volume of 30 μL according to manufacturer instructions for MyTaq^™^ HS DNA polymerase (Bioline, Boston, MA, USA). Amplification products were sequenced by Sanger sequencing at the Australian Genome Research Facility Ltd (http://www.agrf.org.au/). Most primer pairs amplified expected PCR products from both cvs. Gladius and Chinese Spring except for those amplifying the *TaNAS3‐A*,* TaNAS1‐A*,* TaNAS7‐A2*,* TaNAS7‐D* and *TaNAS4‐D* genes; in these cases, the primer pairs amplified the PCR products from cv. Chinese Spring only. In the case of the *TaNAS4‐U* gene, the primer pairs amplified a PCR product from cv. Gladius only.

### Phylogenetic analysis

Phylogenetic analysis of the 20 full‐length *TaNAS* coding sequences was performed in Geneious Pro 5.6.6 using the Geneious plugin PhyML (Guindon *et al*., 2010) and a Kimura 2‐parameter substitution model with bootstrap value of 1000. Published coding sequences of 3 rice *OsNAS* (*OsNAS1*, LOC_Os03 g19427.1; *OsNAS2*, LOC_Os03 g19420.1; *OsNAS3,* LOC_Os07 g48980.1), 10 barley *HvNAS* (Table S1) and nine maize *ZmNAS* (Table S2) genes were included in the phylogenetic analysis.

### Quantitative reverse transcription PCR (qRT‐PCR) analysis of *TaNAS* genes

Preparation of a wheat cv. Chinese Spring cDNA library representing a range of tissues and developmental stages has been described previously, where each cDNA was generated from pooled RNA representing 7–10 individual plants (Schreiber *et al*., 2009). We prepared a cv. Gladius cDNA library; the snap‐frozen root and shoot tissues harvested from individual 4‐week‐old hydroponically grown plants were pulverized and total RNA was extracted from 90 to 100 mg of ground plant material using TRIzol^®^ Reagent (Life Technologies, Carlsbad, CA, USA) according to manufacturer instructions with the following modifications: 250 μL of a 0.8 m sodium citrate and 1.2 m sodium chloride solution was added at the precipitation step and two final washes were performed. Approximately 2 μg of RNA was treated with DNase I (Invitrogen, Carlsbad, CA, USA) in 20 μL reactions to remove genomic DNA contamination and oligo‐dT‐primed reverse transcription was performed following manufacturer instructions (Tetro cDNA synthesis Kit, Bioline). Reactions were performed in a final volume of 20 μL and then diluted 1 : 10. The cDNA samples (cv. Gladius) were verified as genomic DNA free through lack of amplification in PCRs using primer sets specific to the noncoding microsatellite *Xcfb43* as well as a noncoding region of the *TaWIN1* gene (Table S3).

Subgenome‐specific qRT‐PCR primer pairs for each *TaNAS* gene were designed using the same methodology as described under ‘Gene Sequencing’ (Table 2). The specificity of each qRT‐PCR primer pair was determined by melt curve analysis and sequencing. For each primer pair, primer efficiency was calculated using the formula 10^(−1/m)^ − 1, where m corresponds to the slope of the standard curve generated using triplicate ten‐fold serial dilutions (10^1^–10^7^) of purified template for each primer pair (Table 2). The qRT‐PCR analysis of *TaNAS* gene expression was performed using the cDNA libraries of cvs. Chinese Spring and Gladius according to the protocols described in Vandesompele *et al*. (2002). The geometric mean of the three housekeeping genes *TaCyclophilin, TaGAPDH* and *TaEFA* was used as a normalization factor to normalize the expression of the *TaNAS* genes as described in Schreiber *et al*. (2009). A one‐way ANOVA followed by a Tukey's test was performed within days (Days 0, 1, 5 and 7) and between treatments (+Fe and ‐Fe) to identify significant differences in the cv. Gladius qRT‐PCR experiments. Normalization of cv. Gladius qRT‐PCR gene expression was performed independently for the root and shoot tissues (Figure S2).

**Table 2:** Quantitative reverse transcription PCR (qRT‐PCR) analysis of the 21 *TaNAS* genes. The table provides gene name, forward and reverse primer sequences, PCR product length (bp) and the qRT‐PCR efficiency

Gene name	Forward primer sequence (5′‐3′)	Reverse primer sequence (5′‐3′)	PCR product length	qRT‐PCR efficiency
*TaNAS1‐A*	AAGTCGACGCGCTCTTCACC	GCTCAGGTTGATGTAGTTGTCA	230	0.98
*TaNAS1‐B*	AAGTCGACGCGCTCTTCACG	GCTCAGGTTGATGTAGTTGTCG	230	–
*TaNAS2‐A*	GTGCGTACGACGTGGTCTTC	GCTCTTTGGTGGCCAACTCT	388	0.92
*TaNAS2‐D1*	CTCTTTGGCGGCCAGCTCT	CACCCCGAAGGTGAGGTG	189	–
*TaNAS2‐D2*	CTCTTTGGTGGCCAACTCGT	CACCCCGAAGGTGAGGTC	189	0.99
*TaNAS3‐A*	GGTTCCTGTACCCGATCGTA	TGTCACCATCTCACCAAACC	221	0.94
*TaNAS3‐B*	GCTGCTGAGCTCGCTACATA	CCTCTCTCTTGTGGGTCACG	391	0.97
*TaNAS4‐A*	CCTTCGACAACCCTCTGGAC	ACATGGTGTCGGGCAGGT	198	1.03
*TaNAS4‐U*	ACTACGACCTGTGTGGCACA	ATCACCTTCGCCTTGTCCT	187	0.97
*TaNAS4‐D*	ACGTCGACGCGCTCTTCACT	ATGCCGAGGTGATCCAGA	197	1.01
*TaNAS5‐B*	GCGGGTTCCTATACCCGAT	TGCATGTCCTTCGACTTGTG	130	0.96
*TaNAS6‐A*	CTGGAGGCGCACTACTCCTA	GTCGGCGGTGTGGAAAGA	330	0.97
*TaNAS6‐B*	CACCACCTCGCCATCTTC	ACGTCAGCGGTGTGGAAC	283	0.95
*TaNAS6‐D*	GACGACGACGTGGTGAACT	GATCAGAAGGCCACTTCA	203	0.92
*TaNAS7‐A1*	GTGCGAGCAAGCTGTTCC	AAACTCCTCTCTCTTCTGGCTCA	473	0.91
*TaNAS7‐A2*	TGCCCTCGCTCAGCCCGTG	AGTAGTGCGCCTCCAGCTTC	174	0.98
*TaNAS7‐D*	TCAGCAAGCTGGAGTACGAG	CATGCATGTCGTTGGACTTT	528	0.88
*TaNAS8‐U*	GTCCAAAAGATCACCGGACT	GAGTCCCTCCCGCATTTTCT	168	0.9
*TaNAS9‐A*	GCCTCCTTCGACAACTACGA	GGAACACCACGTCGTAGC	154	1.07
*TaNAS9‐B*	CCTGTACCCGGTGGTGAA	CATGGCCATCTCCTTCATCT	265	0.92
*TaNAS9‐D*	GCCTCCTTCGACAACTACGA	ACCACCGGGTACAGGAAG	287	0.92

## Supporting information


**Figure S1.** Morphology of hydroponically grown bread wheat cv. Gladius plants.
**Figure S2.** Expression profiles of the three control genes prior to normalization–*TaCyclophilin* (black), *TaGAPDH* (grey) and *TaEFA* (white) in bread wheat cv. Gladius (a) shoot and (b) root tissues.
**Figure S3.** Relative expression of seven *TaNAS* genes in shoot tissues of bread wheat cv. Gladius under Fe sufficient/deficient conditions.
**Figure S4.** Bread wheat cv. Chinese Spring contains a premature stop codon in the *TaNAS5‐B* coding sequence.

**Table S1.** Barley *HvNAS* genes used in phylogenetic analyses.
**Table S2.** The maize *ZmNAS* genes used in phylogenetic analyses.
**Table S3.** Sequence of PCR primers used to screen for genomic DNA contamination in cv. Gladius cDNA samples.
**Table S4.** Prediction of subcellular localization peptides for TaNAS proteins.
**Table S5.** Percentage nucleic acid identity between the 20 full length *TaNAS* genes.
