# Supplementary material for: Identification and molecular characterization of the nicotianamine synthase gene family in bread wheat
Source: Plant Biotechnol J. 2016 Jun 20;14(12):2228–39. doi: 10.1111/pbi.12577 (PMC5103229; doi:10.1111/pbi.12577)
Supplement: Supplementary file 1 — Figure S1. Morphology of hydroponically grown bread wheat cv. Gladius plants. Figure S2. Expression profiles of the three control genes prior to normalization–TaCyclophilin (black), TaGAPDH (grey) and TaEFA (white) in bread wheat cv. Gladius (a) shoot and (b) root tissues. Figure S3. Relative expression of seven TaNAS genes in shoot tissues of bread wheat cv. Gladius under Fe sufficient/deficient conditions. Figure S4. Bread wheat cv. Chinese Spring contains a premature stop codon in the TaNAS5‐B coding sequence. [file PBI-14-2228-s002.docx]

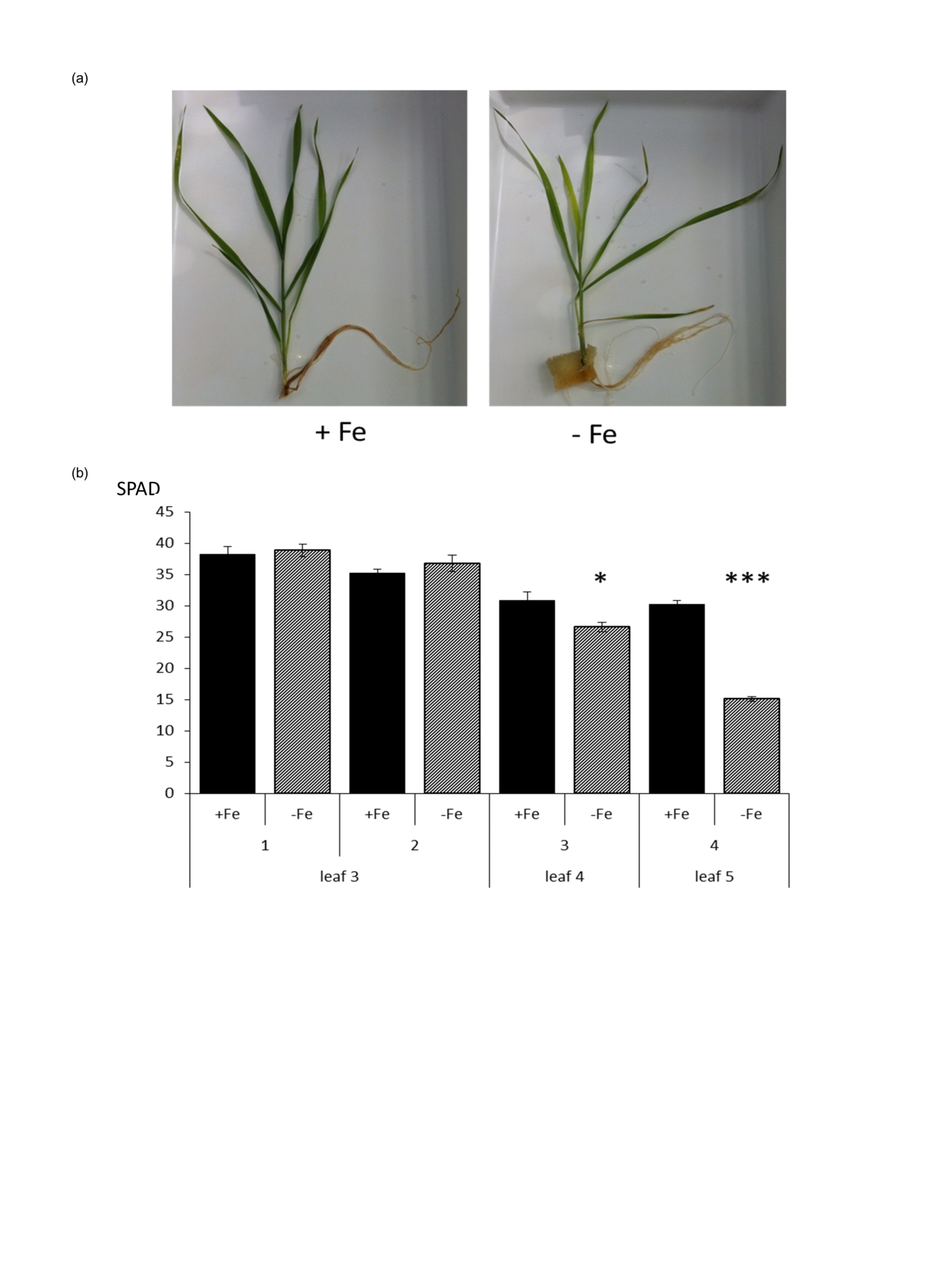


**Figure S1.** Morphology of hydroponically grown bread wheat cv. Gladius plants. (**a)** Representative plants after 7 days growth in hydroponic solution with Fe (left) and hydroponic solution lacking Fe (right). (**b)** SPAD units of leaf chlorophyll content (y-axis) at four time points of the 7 day treatment: day 0 (1), day 1 (1), day 5 (3) and day 7 (4). Black and grey bars correspond to Fe sufficient and deficient treatments, respectively, n=5. Emergence of leaves 3, 4 and 5 is indicated below the time points. Asterisks indicate statistically significant differences for the effect of condition (+Fe and –Fe) at each time point (one-way ANOVA, Tukey test,* p<0.05, *** p<0.001).

**
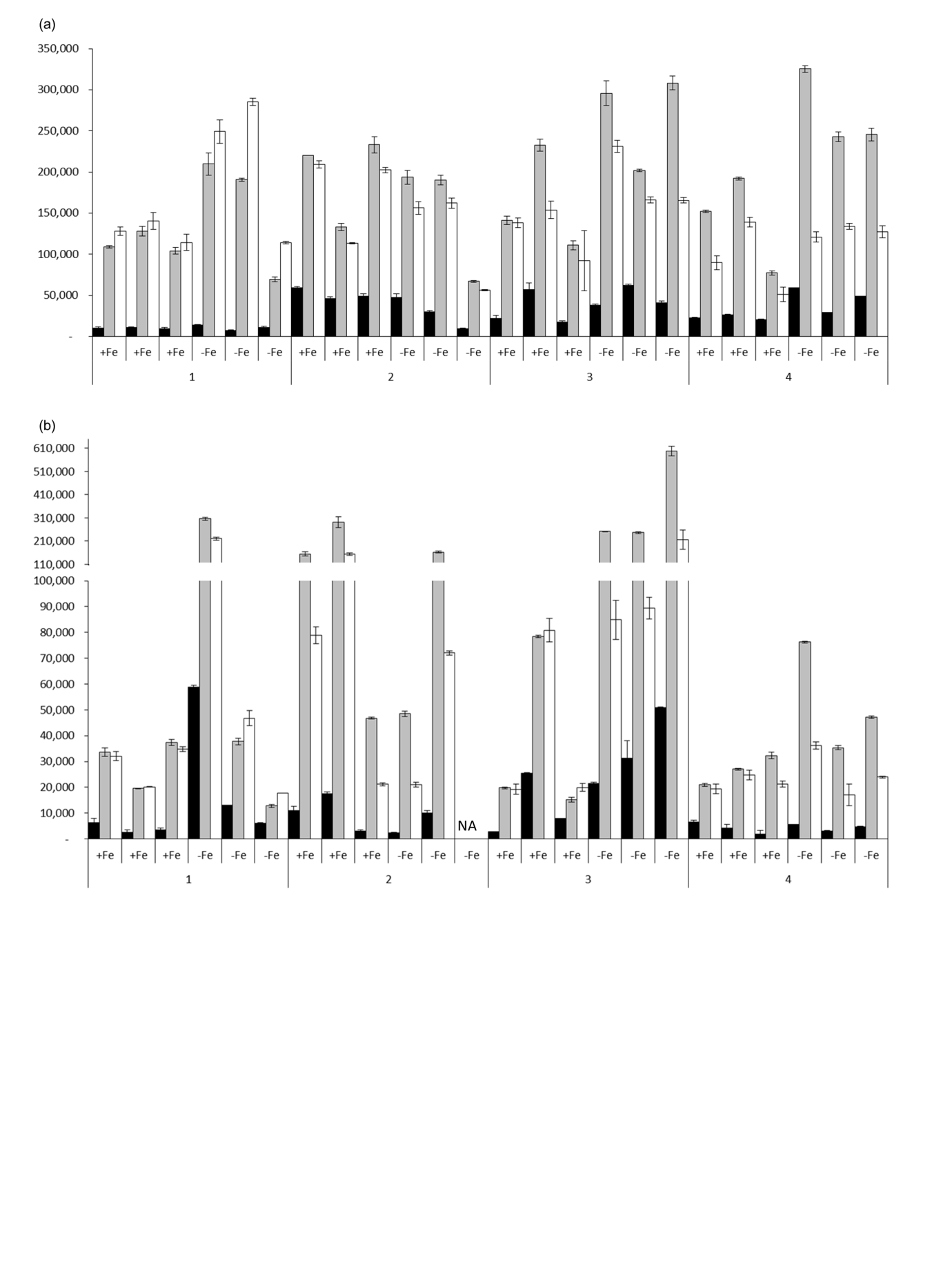
Figure S2.** Expression profiles of the three control genes prior to normalization - *TaCyclophilin* (black), *TaGAPDH* (grey) and *TaEFA* (white) in bread wheat cv. Gladius (**a**) shoot and (**b**) root tissues. Gene expression in plants grown under Fe sufficient (+Fe) or Fe deficient (-Fe) conditions is presented at four time points of the 7 day treatment: day 0 (1), day 1 (1), day 5 (3) and day 7 (4). Units on the y-axis indicate mRNA copies per µg of total RNA. Error bars indicate SEM of three technical replicates.


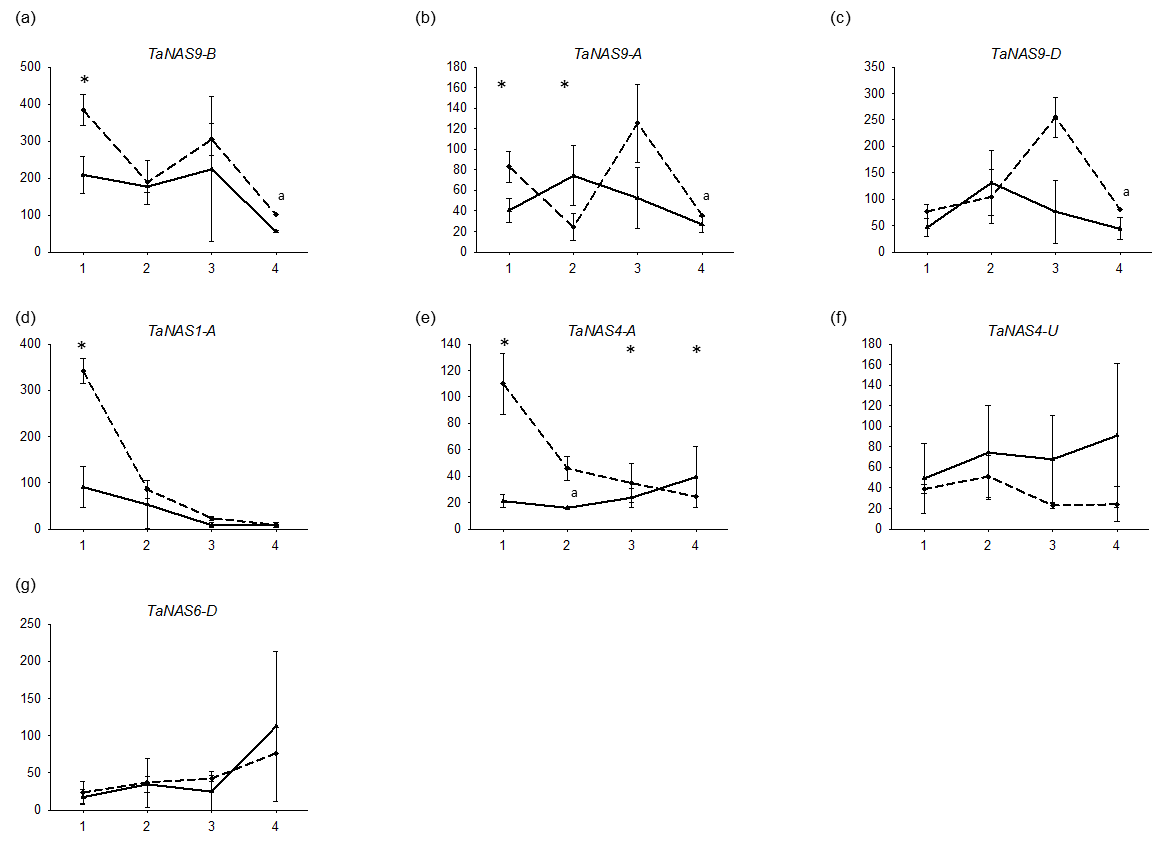


**Figure S3.** Relative expression of 7 *TaNAS* genes in shoot tissues of bread wheat cv. Gladius under Fe sufficient/deficient conditions. Gene expression in plants grown under Fe sufficient (dashed line) or Fe deficient (solid line) conditions is presented at four time points of the 7 day treatment: day 0 (1), day 1 (1), day 5 (3) and day 7 (4). Units on the y-axis indicate normalized mRNA copies per µl of cDNA. Error bars indicate SEM of three biological replicates for time points 1, 2, 3 and 4. Asterisks indicate statistically significant differences for the effect of condition (+Fe and –Fe) at each time point (One-way ANOVA, Tukey test,* = p value ≤0.05, ^a^ = Error bars omitted due to undetectable transcript levels in at least one biological replicate).


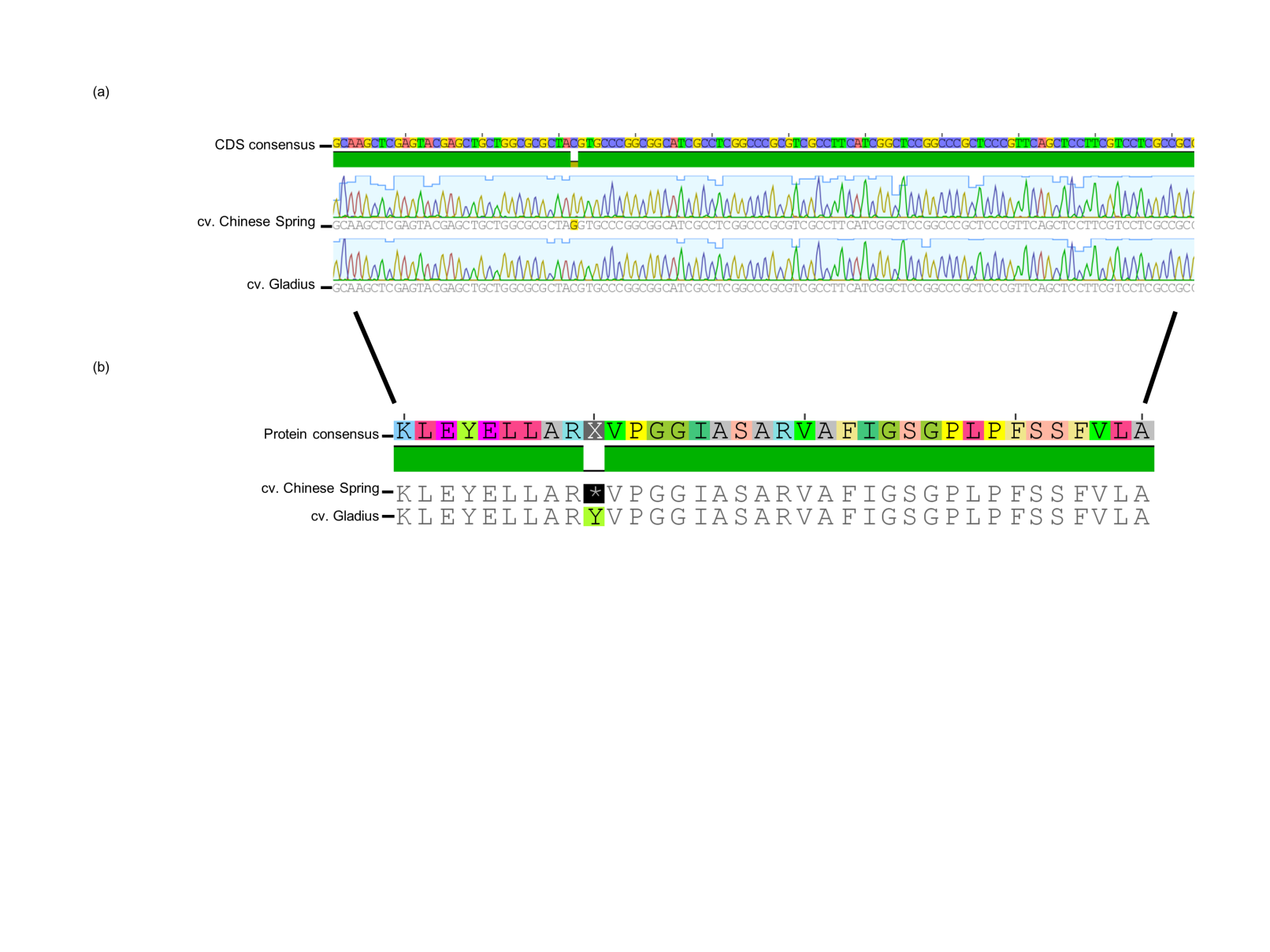


**Figure S4.** Bread wheat cv. Chinese Spring contains a premature stop codon in the *TaNAS5-B* coding sequence. **(a)** Alignment of nucleotides 455 to 566 bp of the *TaNAS5-B* CDS with corresponding genomic sequence from cvs. Chinese Spring and Gladius. A G→C SNP at nucleotide 486 of cv. Chinese Spring (highlighted in yellow) creates a premature TAG stop codon in the CDS sequence. **(b)** Alignment of amino acids 153 to 188 of the TaNAS5-B protein in cvs. Chinese Spring and Gladius showing the premature stop codon in cv. Chinese Spring at amino acid 162.
