# Supplementary material for: Identification and molecular characterization of the nicotianamine synthase gene family in bread wheat
Source: Plant Biotechnol J. 2016 Jun 20;14(12):2228–39. doi: 10.1111/pbi.12577 (PMC5103229; doi:10.1111/pbi.12577)
Supplement: Supplementary file 2 — Table S1. Barley HvNAS genes used in phylogenetic analyses. Table S2. The maize ZmNAS genes used in phylogenetic analyses. Table S3. Sequence of PCR primers used to screen for genomic DNA contamination in cv. Gladius cDNA samples. Table S4. Prediction of subcellular localization peptides for TaNAS proteins. Table S5. Percentage nucleic acid identity between the 20 full length TaNAS genes. [file PBI-14-2228-s001.docx]

**Supporting information**

**Table S1.** Barley *HvNAS* genes used in phylogenetic analyses. The gene name utilized in the manuscript, the chromosome location, NCBI GenBank identifier and source reference is provided for each gene.

| Gene name | Chromosome location | GenBank ID | Reference |
| --- | --- | --- | --- |
| *HvNAS1* | 6HS | AB010086 | Higuchi et al., 1999b |
| *HvNAS2* | 4HL | AB011265 | Higuchi et al., 1999b |
| *HvNAS3* | 4HL | AB011264 | Higuchi et al., 1999b |
| *HvNAS4* | 6HL | AB011266 | Higuchi et al., 1999b |
| *HVNAS5-1* | 2HS | AB011267 | Higuchi et al., 1999b |
| *HVNAS5-2* | 2HS | AB011268 | Higuchi et al., 1999b |
| *NASHOR1a* | 4HL | AF136941 | Herbik et al., 1999 |
| *HvNAS7* | 6HL | AB019525 | NCBI |
| *NASHOR1b* | 6HL | AB011269 | Perovic et al., 1999 |
| *NASHOR2* | 2HS | AF136942 | Herbik et al., 1999 |

**Table S2.** The maize *ZmNAS* genes used in phylogenetic analyses. The gene name utilized in the manuscript and the corresponding gene ID from Zhou et al., (2013b) is provided for each gene.

| Gene name | Gene ID |
| --- | --- |
| *ZmNAS1;1* | GRMZM2G385200 |
| *ZmNAS2;1* | GRMZM2G030036 |
| *ZmNAS3* | GRMZM2G478568 |
| *ZmNAS2;2* | GRMZM2G124785 |
| *ZmNAS4* | GRMZM2G439195 |
| *ZmNAS5* | GRMZM2G050108 |
| *ZmNAS6;1* | GRMZM2G704488 |
| *ZmNAS6;2* | AC233955.1_FGT003 |
| *ZmNAS1;2* | GRMZM2G312481 |

**Table S3.** Sequence of PCR primers used to screen for genomic DNA contamination in cv. Gladius cDNA samples. The *Xcfb43* primers amplify the non-coding microsatellite *Xcfb43* and the *TaWIN1* primers span a non-coding region (intron) of the *TaWIN1* gene.

| Name | Type | Source | Forward primer sequence 5’ to 3’ | Reverse primer sequence 5’ to 3’ | PCR product |
| --- | --- | --- | --- | --- | --- |
| *Xcfb43* | microsatellite | ^1^BAC sequence: 3B_005_N23_FM1 | AGCTTCCTCAAGAGCCATC | CCAAGTAAGCAAGAGGATGAG | DNA (327 bp) |
| *TaWIN1* | gene | GenBank locus: AB042193 | GGACAGCTTAGGCGAGGAAT | GCTGGGGCTTCCTTAATCTC | cDNA (126 bp) / DNA (213 bp) |

^1^ Bacterial artificial chromosome (BAC) sequence from GrainGenes database - <http://wheat.pw.usda.gov/GG2/>

**Table S4.** Prediction of subcellular localization peptides for TaNAS proteins. For each *TaNAS* gene the table provides protein length (Len) and the scores for chloroplast transit peptide (cTP), mitochondrial targeting peptide (mTP), secretory pathway signal peptide (SP) and other subcellular localization including cytoplasmic, nuclear and peroxisomal peptides (Other) using the recommended protocol for TargetP 1.1 as described by Emanuelsson et al., (2007). Significant scores are summarized in the predicted localization (Loc) column for proteins containing mitochondrial targeting (M), chloroplast transit (C) or secretory pathway signal (S) peptides. The TargetP1.1 prediction scores (RC) for reliability and confidence are also provided.

| Gene name | Len | cTP | mTP | SP | Other | Loc | RC |
| --- | --- | --- | --- | --- | --- | --- | --- |
| *TaNAS1-A* | 281 | 0.357 | 0.076 | 0.076 | 0.523 | _ | 5 |
| *TaNAS1-B* | 287 | 0.248 | 0.064 | 0.113 | 0.562 | _ | 4 |
| *TaNAS2-A* | 327 | 0.143 | 0.023 | 0.414 | 0.412 | S | 5 |
| *TaNAS2-D1* | 330 | 0.235 | 0.027 | 0.241 | 0.431 | _ | 5 |
| *TaNAS2-D2* | 330 | 0.199 | 0.028 | 0.27 | 0.496 | _ | 4 |
| *TaNAS3-A* | 331 | 0.15 | 0.083 | 0.244 | 0.389 | _ | 5 |
| *TaNAS3-B* | 331 | 0.183 | 0.078 | 0.23 | 0.394 | _ | 5 |
| *TaNAS4-A* | 334 | 0.324 | 0.057 | 0.173 | 0.473 | _ | 5 |
| *TaNAS4-U* | 334 | 0.331 | 0.06 | 0.172 | 0.474 | _ | 5 |
| *TaNAS4-D* | 334 | 0.214 | 0.047 | 0.291 | 0.524 | _ | 4 |
| *TaNAS5-B* | 376 | 0.053 | 0.604 | 0.039 | 0.024 | M | 3 |
| *TaNAS6-A* | 353 | 0.848 | 0.194 | 0.003 | 0.051 | C | 2 |
| *TaNAS6-B* | 331 | 0.126 | 0.065 | 0.28 | 0.627 | _ | 4 |
| *TaNAS6-D* | 385 | 0.895 | 0.066 | 0.003 | 0.111 | C | 2 |
| *TaNAS7-A1* | 331 | 0.133 | 0.081 | 0.197 | 0.616 | _ | 3 |
| *TaNAS7-A2* | 331 | 0.119 | 0.074 | 0.258 | 0.53 | _ | 4 |
| *TaNAS7-D* | 331 | 0.163 | 0.116 | 0.118 | 0.601 | _ | 3 |
| *TaNAS9-A* | 341 | 0.216 | 0.023 | 0.115 | 0.722 | _ | 3 |
| *TaNAS9-B* | 341 | 0.173 | 0.025 | 0.146 | 0.726 | _ | 3 |
| *TaNAS9-D* | 341 | 0.232 | 0.025 | 0.092 | 0.783 | _ | 3 |

**Table S5.** Percentage nucleic acid identity between the 20 full length *TaNAS* genes. Groups of homeologous genes with at least 94% identity are highlighted in common colour.

| Clade/  Subgroup |  | *TaNAS1-A* | *TaNAS1-B* | *TaNAS2-A* | *TaNAS2-D1* | *TaNAS2-D2* | *TaNAS3-A* | *TaNAS3-B* | *TaNAS4-A* | *TaNAS4-U* | *TaNAS4-D* | *TaNAS5-B* | *TaNAS6-A* | *TaNAS6-B* | *TaNAS6-D* | *TaNAS7-A1* | *TaNAS7-A2* | *TaNAS7-D* | *TaNAS9-A* | *TaNAS9-B* | *TaNAS9-D* |
| --- | --- | --- | --- | --- | --- | --- | --- | --- | --- | --- | --- | --- | --- | --- | --- | --- | --- | --- | --- | --- | --- |
| 1/1 | *TaNAS1-A* | 100 |  |  |  |  |  |  |  |  |  |  |  |  |  |  |  |  |  |  |  |
|  | *TaNAS1-B* | 91.8 | 100 |  |  |  |  |  |  |  |  |  |  |  |  |  |  |  |  |  |  |
| 1/3 | *TaNAS2-A* | 67.1 | 67.6 | 100 |  |  |  |  |  |  |  |  |  |  |  |  |  |  |  |  |  |
|  | *TaNAS2-D1* | 66.5 | 67.3 | 96.3 | 100 |  |  |  |  |  |  |  |  |  |  |  |  |  |  |  |  |
| 1/3 | *TaNAS2-D2* | 66.7 | 67.2 | 96.1 | 98 | 100 |  |  |  |  |  |  |  |  |  |  |  |  |  |  |  |
|  | *TaNAS3-A* | 65.9 | 66.8 | 78.5 | 78.4 | 78.9 | 100 |  |  |  |  |  |  |  |  |  |  |  |  |  |  |
|  | *TaNAS3-B* | 65.5 | 66.4 | 78.5 | 78.4 | 79 | 94.6 | 100 |  |  |  |  |  |  |  |  |  |  |  |  |  |
| 1/3 | *TaNAS4-A* | 65.3 | 66.5 | 79.1 | 79.2 | 79.7 | 90.7 | 90.5 | 100 |  |  |  |  |  |  |  |  |  |  |  |  |
| 1/3 | *TaNAS4-U* | 64.4 | 65.5 | 77.8 | 78 | 78.4 | 89.5 | 89.4 | 93.9 | 100 |  |  |  |  |  |  |  |  |  |  |  |
|  | *TaNAS4-D* | 64.5 | 65.6 | 78.2 | 78.2 | 78.6 | 90.2 | 90.2 | 94.7 | 96.8 | 100 |  |  |  |  |  |  |  |  |  |  |
|  | *TaNAS5-B* | 66.6 | 67.4 | 80.3 | 80 | 80.8 | 90.7 | 89.9 | 91.1 | 90.4 | 90.9 | 100 |  |  |  |  |  |  |  |  |  |
| 1/3 | *TaNAS6-A* | 73.2 | 74.1 | 80.8 | 81.1 | 81.2 | 90.8 | 89.9 | 89 | 88.2 | 88.6 | 84.6 | 100 |  |  |  |  |  |  |  |  |
|  | *TaNAS6-B* | 66.6 | 67.6 | 80.5 | 80.6 | 81.4 | 91.6 | 90.8 | 89.8 | 90.5 | 90.2 | 92.5 | 95.7 | 100 |  |  |  |  |  |  |  |
|  | *TaNAS6-D* | 67.4 | 68.5 | 81.8 | 81.9 | 82.5 | 92.3 | 91.4 | 90.6 | 90.1 | 90.5 | 86.5 | 96.8 | 97.7 | 100 |  |  |  |  |  |  |
| 1/2 | *TaNAS7-A1* | 66.6 | 67.8 | 81.7 | 81.5 | 82.2 | 91 | 90.4 | 89.4 | 89.4 | 89.3 | 90.9 | 93.5 | 94.7 | 95.5 | 100 |  |  |  |  |  |
|  | *TaNAS7-A2* | 66.2 | 67.7 | 81 | 80.8 | 81.5 | 89.9 | 89.6 | 89 | 88.7 | 88.7 | 90.9 | 92.1 | 93.5 | 94.4 | 95.4 | 100 |  |  |  |  |
|  | *TaNAS7-D* | 66.2 | 67.4 | 80.7 | 80.6 | 81.3 | 90.5 | 89.9 | 88.8 | 88.8 | 89 | 91 | 92.9 | 94.5 | 95.3 | 96.5 | 96.3 | 100 |  |  |  |
| 2/- | *TaNAS9-A* | 60.9 | 62.2 | 70.5 | 70.8 | 71.3 | 71.8 | 71.6 | 72.9 | 72.2 | 72.1 | 74 | 73.3 | 72.2 | 72.9 | 73.2 | 73 | 72.3 | 100 |  |  |
|  | *TaNAS9-B* | 60.4 | 61.7 | 70.5 | 70.8 | 71.3 | 71.5 | 71.1 | 72.6 | 72.1 | 72 | 73.8 | 72.7 | 72.1 | 72.7 | 73.3 | 73.2 | 72.4 | 98.4 | 100 |  |
|  | *TaNAS9-D* | 60.7 | 61.9 | 70.2 | 70.5 | 71.1 | 71.9 | 71.6 | 72.6 | 72 | 72 | 73.9 | 73 | 72.3 | 73 | 73.4 | 73.3 | 72.5 | 97.9 | 97.6 | 100 |
